# Growing Pains

**Published:** 1911-12

**Authors:** L. B. Clarke

**Affiliations:** Atlanta, Ga.


### Journal-Record of Medicine

Successor to Atlanta Medical and Surgical Journal, Established 1855, and Southern Medical Record, Established 1870.

OWNED BY THE ATLANTA MEDICAL JOURNAL CO.

**Published Monthly**

*Official Organ Fulton County Medical Society, State Examining Board, Presbyterian Hospital, Atlanta, Birmingham and Atlantic Railroad Surgeons' Association, Chattahoochee Valley Medical and Surgical Association, Etc.*

EDGAR G. BALLENGER., M. D., Editor.
BERNARD WOLFF, M. D., Supervising Editor.
A. W. STIRLING, M. D., C. M., D. P. H., J. S. HURT, B. Ph., M. D.
GEO. M. NILES, M. D., W. J. LOVE, M. D., (Ala.); Associate Editors.
E. W. ALLEN, Business Manager.

COLLABORATORS

Dr. W. F. WESTMORLAND, General Surgery.
F. W. McRAE, M. D., Abdominal Surgery.
H. F. HARRIS, M. D., Pathology and Bacteriology.
MICHAEL HOKE, M. D., Orthopedic Surgery.
CYRUS W. STRICKLER, M. D., Legal Medicine and Medical Legislation.
E. C. DAVIS, A. B., M. D., Obstetrics.
E. G. JONES, A. B., M. D., Gynecology.
R. T. DORSEY, Jr., B. S. M. D., Medicine.
L. M. GAINES, A. B., M. D., Internal Medicine.
J. N. LeCONTE, M. D., Disease of the Stomach and Intestines.
L. B. CLARKE, M. D., Pediatrics.
EDGAR PAULIN, M. D., Opsonic Medicine.
THEODORE TOEPEL, M. D., Mechano Therapy.
R. R. DALY, M. D., Medical Society.
A. W. STIRLING, M. D., etc., Diseases of the Eye, Ear, Nose and Throat.
BERNARD WOLFF, M. D., Diseases of the Skin.
E. G. BALLENGER, M. D., Diseases of the Genito-Urinary Organs.

VOL. LVIII. DECEMBER 1911 NO. 9.

### GROWING PAINS.

BY L. B. CLARKE, M. D., ATLANTA, GA.

There are still many relics of the gloom of what may be termed Medical Dark Ages, which have not yet been dispelled by modern scientific research, and among these, none stand out more pre-eminent than "Growing Pains." It is surprising with what tenacity some of these old superstitions hold on, and how difficult they are to dissipate, but it is nevertheless true.

Growing pains still occupy a hitherto unsuccessfully assailed position, in corners not necessarily dark; but in many instances found where a deal of light should have been shed.

Time was, even so late as the childhood of the average middle-aged adult of to-day, when "Growing Pains" were as absolutely necessary as the stumped toe or the bruised shin or "Hives," along the path of infancy and childhood, and as one estimable lady remarked to me recently, "How much pain it had caused her to grow."

How frequently still, does some fond parent sit up half the night rubbing the legs of her child with witch hazel or alcohol, or some other local application, in her vain attempts to relieve what she terms "growing pains," totally unaware of the true nature of this insidious form of rheumatism. Insidious is a well-advised term in such a case, for the very reason that unrecognized, it continues to develop in the victim unheeded and untrammeled, until the day shall arrive at which one or more of the many complications of rheumatism are manifest.

Rheumatism was formerly believed to be very rare in early life, but latter day information has taught us that it is fairly common, and that growing pains are one of its ordinary manifestations. He who awaits the clinical symptoms of acute articular rheumatism, as seen in adult life, and must have an inflammation of a joint in order to diagnose "rheumatism" in a child, will overlook many cases of the disease.

Indeed, modern pediatrics, as wlll be later ponted out, associates certain other diseases so closely with rheumatism, and among them chorea, that in chorea of rheumatic origin, the chorea is quite commonly the first evidence of the existence of rheumatism. This brings us to the point at which the growing pain assumes the important position, then, that it should occupy, namely, the consideration of a certain chain of diseases that are intimately connected with rheumatism in childhood, sometimes as complications, in other instances sequelae, and in a certain number of cases existing conjointly or reversing their positions.

The chain to which I have referred, are growing pains, tonsillitis, rheumatism, plus or not arthritis, chorea and endoarditis. The peculiarity surrounding these associated diseases is the readily traced fact that they have no regular order of sequence, and are diversified in their appearance in the patient as a game of chance. In some cases we will find, as previouslv alluded to:—chorea to be the first manifestation of rheumatism; the rheumatism developing later, stamps the nature of the chorea. Following the chorea will be an endocarditis. In other instances, there may be no evidenes of rheumatism from the standpoint of pain, and still the endo-carditis. In other instances, the chorea and the endocarditis seem to have appeared simultaneously; in still other instances thec horea follows the endocarditis. In many of these repeated attacks of tonsilitis have antedated the subsequent chorea and endocarditis, and in many of these, there is still no history of what is ordinarily termed rheumatism, except the "growing pains," which have been forgotten.

The essayist has had opportunity to analyze quite a number of these cases, having become much interested in the unremitting regularity which many or all of this chain of diseases had appeared in the patient in some or other order.

Some cases have gone through the entire gamut, while others have managed to escape one and sometimes two of the enumerated conditions.

Among my patients is a young girl of 19. I have treated her in two attacks of acute rheumatism, the prominet symptoms of which were, temperature, pain in the muscles, and a general stiffness of joints, but never the slightest evidence of articular rheumatism or enlargement. The pain and the stiffness were migratory in character, which would at once stamp the disease were there no other evidences. These attacks were about two years apart; each atack was ushered in with general sore throat, and in the first there was an additional tonsilitis with a deposit so suspicious in its nature, that a culture was taken, which proved negative. This patient was a perfect martyr to tonsilitis. She had suffered from the disease for years with recurrent attacks almost each winter. Going back into her history at the time of the first attack, it was ascertained that she had had chorea. Some time after the chorea, endocarditis developed, the development of which had been ascribed to an exceedingly narrow escape from being crushed by a street car, the resultant shock and the fright, it had been suggested, producing the disease. The mother, a very intellectual woman, was astounded at the suggestion that the chorea and the endocarditis and the tonsillitis and the subsequent manifest rheumatism were associated, and then came the usual "growing pains" history.

A little girl of 11 years was brought in to be treated for "nervousness." A glance was sufficient to diagnose chorea. It was impossible for her to sit still sufficiently long for a heart examination, at that time. Later, the heart was found involved. A short time afterward, within a few weeks, she was treated through an acute rheumatism, asfisociated with an acutee ndocarditis, both of which appeared simultaneously. There is an extensive "growing pain" history in the children of this particular family.

A boy of 13 years developed chorea. He had previously undergone four or five attacks of tonsilitis. There were no other evidences of rheumatism, nor did the heart become involved at the time or later, but still, the growing pains.

The purpose andi ntent of this paper is not to discuss any of the many diseases which have been mentioned, but to bring forward their deadly and destructive nature, and their association with rheumatism even though the rheumatism be not recognized; that growing pains, so called, are rheumatic and to pass these conditions unnoticed and untreated, with the too often statement that the child will outgrow them, is but to invite and encourage the advent of disastrous consequences.

An additional purpose is to place before you the consideration of this associated chain of diseases in a new light, namely, knowing the uncertain and irregular and extraordinary manner in which any one of them may be the initial disease, and any one or more of them prove complications or sequelae or neither, and that their positions in the rheumatic diathesis so interchangeable and reversible, shall we not yet conclude that growing pains, tonsilitis, chorea and rheumatic endocarditis are simply symptoms of rheumatism?

Peters Bldg., Atlanta, Ga.

